# Methanol extract of *Muntingia calabura* leaves attenuates CCl_4_-induced liver injury: possible synergistic action of flavonoids and volatile bioactive compounds on endogenous defence system

**DOI:** 10.1080/13880209.2019.1606836

**Published:** 2019-05-08

**Authors:** Zainul Amiruddin Zakaria, Nur Diyana Mahmood, Maizatul Hasyima Omar, Muhammad Taher, Rusliza Basir

**Affiliations:** a Laboratory of Halal Science Research, Halal Products Research Institute, Universiti Putra Malaysia, Serdang, Malaysia;; b Integrative Pharmacogenomics Institute (iPROMISE), Universiti Teknologi MARA, Puncak Alam, Malaysia;; c Department of Biomedical Science, Faculty of Medicine and Health Sciences, Universiti Putra Malaysia, Serdang, Malaysia;; d Phytochemistry Unit Herbal Medicine Research Centre, Institute for Medical Research, Kuala Lumpur, Malaysia;; e Department of Pharmaceutical Technology, Kulliyah of Pharmacy International Islamic University Malaysia, Kuantan, Malaysia;; f Department of Human Anatomy, Faculty of Medicine and Health Sciences, Universiti Putra Malaysia, Serdang, Malaysia

**Keywords:** Muntingiaceae, hepatoprotective activity, antioxidant activity, oxidative stress markers, pro-inflammatory mediators, phytoconstituents, UHPLC-ESI, GCMS

## Abstract

**Context:***Muntingia calabura* L. (Muntingiaceae) exerts antioxidant and anti-inflammatory activities, thus, it might be a good hepatoprotective agent.

**Objective:** This study investigates the effect of methanol extract of *M. calabura* leaves (MMCL) on hepatic antioxidant and anti-inflammatory activities in CCl_4_-induced hepatotoxic rat.

**Materials and methods:** Sprague Dawley rats (*n* = 6) were treated (p.o.) with 10% DMSO (Groups 1 and 2), 50 mg/kg *N*-acetylcysteine (Group 3) or, 50, 250, or 500 mg/kg MMCL (Groups 4–6) for 7 consecutive days followed by pretreatment (i.p.) with vehicle (Group 1) or 50% CCl_4_ in olive oil (v/v) (Groups 2–6) on day 7th. Plasma liver enzymes and hepatic antioxidant enzymes and pro-inflammatory cytokines concentrations were measured while liver histopathology was examined.

**Results:** MMCL, at 500 mg/kg, significantly (*p* < 0.05) ameliorated CCl_4_-induced hepatotoxicity by decreasing the plasma level of alanine transaminase (429.1 versus 168.7 U/L) and aspartate transaminase (513.8 versus 438.1 U/L) as well as the tissue level of nitric oxide (62.7 versus 24.1 nmol/g tissue). At 50, 250, or 500 mg/kg, MMCL significantly (*p* < 0.05) reduced the tumour necrosis factor α (87.8 versus 32.7 pg/mg tissue), interleukin-1β (1474.4 versus 618.3 pg/mg tissue), and interleukin-6 (136.7 versus 30.8 pg/mg tissue) while increased the liver catalase (92.1 versus 114.4 U/g tissue) and superoxide dismutase (3.4 versus 5.5 U/g tissue). Additionally, qualitative phytochemicals analysis showed that MMCL contained gallic acid, ferulic acid, quercetin, and genistein.

**Discussion and conclusions:** MMCL ability to attenuate CCl_4_-induced hepatotoxicity could be helpful in the development of hepatoprotective agents with fewer side effects.

## Introduction

The liver plays a vital role in the metabolism and detoxification of various xenobiotics that could potentially harm the body. However, over-exposure to toxic chemicals that can cause oxidative stress and lipid peroxidation may result in liver damage. Among these chemicals is carbon tetrachloride (CCl_4_), a highly toxic compound causing liver damage through its conversion to free radicals by hepatic microsomal cytochrome P450 (CYP_450_) (Giannini et al. 2005; Manibusan et al. 2007; Ohata et al. 2008). The generation of CCl_3_ radical also inhibits lipoprotein secretion causing steatosis and CCI_3_^−OO^ initiates lipid peroxidation in the liver (Boll et al. 2001).

Currently, modern medicine still has little to offer for the prevention and attenuation of liver diseases and damages (Tukappa et al. 2015). Thus, patients often resort to herbal products as an alternative source of treatment for their ailments. There are several hepatoprotective herbal-based compounds that purportedly possess oxidative stress detoxification properties with minimum or no side effects (Bezenjani et al. 2012). One of the plants reported to possess high anti-inflammatory and antioxidant activities and as a potential source of hepatoprotective compounds is *Muntingia calabura* L. (Muntingiaceae) (Mahmood et al. 2014a). Known to the Malays as *‘kerukup siam’* or *‘ceri kampung’*, *M. calabura* has no medicinal value in Malay folklore medicine, although it has been used in traditional medicine in several Southeast Asian and tropical American countries. Among traditional uses of parts of the tree are for the treatment of gastric ulcer, headache, cold, stomachache, measles, and pimples (Mahmood et al. 2014a).

In accordance with these claims, scientific studies have revealed the vast pharmacological potential of different parts of *M. calabura*. Based on the literature review on *M. calabura* for pharmacological reports between 1991 and 2014, it has been reported to demonstrate cytotoxic, antiproliferative, insecticidal, hypotensive, antinociceptive, cardioprotective, antipyretic, antiplatelet aggregation, antioxidant, anti-inflammatory, antidiabetic, antiulcer, and antibacterial activities with approximately 87 flavonoid-based compounds identified (Mahmood et al. 2014a).

We showed in an earlier study that methanol extract of *M. calabura* leaves (MMCL) (50–500 mg/kg) attenuates paracetamol (PCM)-induced liver injury (Mahmood et al. 2014b). MMCL contains significant amounts of flavonoid, saponins, tannins, and some phenolic compounds, which are known to exert anti-inflammatory and antioxidant activities. It is through these components that MMCL ameliorated PCM-induced liver injury.

At this juncture, it is not known how the MMCL modulates liver metabolism in carbon tetrachloride (CCl_4_)-induced hepatotoxicity. Thus, in this study, we determined the effect of MMCL on CCl_4_-induced hepatotoxicity by estimating antioxidant activities, and release of oxidative stress markers and pro-inflammatory cytokines in the rat model.

## Materials and methods

### Chemicals

Methanol, petroleum ether, and ethyl acetate, the solvents used for plant extraction and for phytochemical analyses including formic acid, and LCMS grade acetonitrile were purchased from MERCK (Selangor, Malaysia). The kits used for the determination of catalase and superoxide dismutase were purchased from Cayman Chemicals (Ann Arbor, MI). HPLC grade water was prepared from distilled water using a Milli-Q-system (Millipore, Waltham, MA). Gallic acid, kaempferol, kaempferol-3-*O*-glucoside, and quercetin were purchased from Sigma (St. Louis, MO), and isoferulic acid from Extrasynthese (Genay, France). All of other solvents and chemicals used in this study were of analytical grade. Stock and working standards for the phytochemical analyses were prepared by dissolving the analytes in 100% methanol and stored at 4 °C until use.

### Collection of plant material and preparation of plant extract

The collection of plant material and the preparation of plant extract have been described in detail elsewhere (Mahmood et al. 2014b). Briefly, the leaves were collected from its natural habitat in Serdang, Selangor, Malaysia, authenticated by a certified botanist (Dr Shamsul Khamis of the Institute of Bioscience (IBS), UPM) based on the previous sample (voucher specimen – SK 2466/14) and deposited in the Herbarium of Institute of Bioscience, Universiti Putra Malaysia (UPM).

The leaves were air-dried for 7 days at room temperature (27 ± 2 °C) taking special care to avoid the formation of moulds on the leaves. The air-dried leaves (500 g) were ground into a coarse powder and then immersed in 10 L methanol [a ratio of 1:20 (w/v)] for 72 h. The supernatant was filtered using cloth filter, cotton wool, and, finally, Whatman No. 1 filter paper. The remaining residue was subjected to the same extraction process twice. The supernatants were pooled and evaporated under reduced pressure at 40 °C to obtain dried crude extract (% yield = 17.9%), which was kept at 4 °C until use.

### Animals

The study protocol was approved by the International Animal Care and Use Committee (IACUC), Faculty of Medicine and Health Sciences (FMHS), UPM (Ethical approval no: UPM/IACUC/AUP- R004/2014). Male Sprague–Dawley rats aged 8–10 weeks and weighing 180–200 g were acquired from the Chenur Supplier, Selangor, Malaysia. The rats were housed in the Animal Holding Unit, FMHS, UPM, at room temperature (27 ± 2 °C) under 70–80% humidity and 12 h light/dark cycle. Food and water were provided *ad libitum*. The handling of rats was according to the guidelines for the care of laboratory animals and the ethical guidelines for investigation of experimental pain in conscious animals, FMHS, UPM. Data processing and making exclusions and decisions were done by experimenters blinded to the study. All sections of this report adhered to the ARRIVE Guidelines for reporting animal research. A completed ARRIVE guidelines checklist is included in Checklist S1.

### Hepatoprotective assay

#### Carbon tetrachloride-induced hepatotoxicity test

Each group was treated accordingly with either 10% DMSO (v/v) in distilled water (vehicle 1; Negative control), 50 mg/kg *N*-acetylcysteine (NAC) or, 50, 250, or 500 mg/kg MMCL once daily for 7 consecutive days (Table 1). Oral administration was chosen to mimic the traditional way of consuming the plant extract while the dose range used was based on the data from the previous acute toxicity study (Balan et al. 2014). The inducer, 1 mL/kg body weight 50% CCl_4_ (v/v) in olive oil, was administered orally on day 7 to all group of animals except Group 1, which received 50% olive oil (v/v) in water (vehicle 2) (used to dissolve CCl_4_) (Kamisan et al. 2014). The rats were weighed at approximately 48 h after the administration of CCl_4_ and then anesthetized under diethyl ether. Approximately 3 mL of blood were collected via cardiac puncture into lithium heparinized tubes for biochemical analysis. The blood samples were centrifuged at 1000 × *g* for 10 min using a refrigerated centrifuge to obtain plasma, which were transferred to clean polypropylene tubes and stored at −80 °C until use. The rats were then sacrificed by cervical dislocation the livers collected, weighed, and washed in ice-cold saline. A section from the median lobe of the liver was fixed in 10% formalin for microscopic analysis and the remainder immediately frozen on dry ice and stored at −80 °C until use. 

**Table 1.** Brief explanation on the experimental design for MMCL-induced hepatoprotective study.

Group		Administration of test solution (p.o.)	Dose (mg/kg)	Administration of inducer of day 7
1	Normal	10% DMSO (vehicle 1)	–	**50%** olive oil (vehicle 2)
2	Negative	10% DMSO (vehicle 1)	–	CCl_4_
3	Positive	NAC	50	CCl_4_
4	Treatment	MMCL	50	CCl_4_
5			250	CCl_4_
6			500	CCl_4_

NAC: *N*-acetylcysteine; Vehicle 1 – used to dissolve NAC and MMCL; Vehicle 2 – used to dissolve CCl_4_.

The rats (*n* = 6) were pretreated with the respective test solutions for 7 days followed by CCl_4_-induced hepatotoxicity assay on day 7.

## Plasma liver enzymes

The plasma alanine aminotransaminase (ALT), aspartate aminotransaminase (AST), and alkaline phosphatase (ALP) concentrations were determined spectrophotometrically by means of the Automatic Chemical Analyzer (Hitachi 902, Tokyo, Japan) using commercial kits (BioMed Diagnostics, White City, OR).

## Liver homogenate antioxidant enzyme, pro-inflammatory cytokines, and nitric oxide concentrations

### Preparation of liver homogenates for antioxidant study

Approximately 100 mg of liver tissue were homogenized in 1 mL ice-cold PBS buffer using a steel homogenizer. The homogenate was further centrifuged at 4000 rpm and 4 °C (Thermo Scientific centrifuge (Thermo Fisher Scientific Co., Waltham, MA), Legend Micro 17 R) for 25 min to obtain supernatant, which was used for the determination of superoxide dismutase (SOD) and catalase (CAT) (Zakaria et al. 2015), nitric oxide (NO) (Malyshev and Shnyra 2003) and interleukin-1β (IL-1β), interleukin-6 (IL-6), and tumour necrosis factor alpha (TNF-α) concentrations (Liu et al. 2016) concentrations.

### Superoxide dismutase

The SOD activity of the liver tissue samples was measured using the standard kit (Cayman Chemical, Ann Arbor, MI) according to the manufacturer’s protocol. Briefly, 10 μL of 0, 0.005, 0.01, 0.02, 0.03, 0.04, or 0.05 U/mL SOD standard or samples was added to each well, followed by 200 μL diluted radical detector. The reactions were initiated with the addition of 20 μL diluted xanthine oxidase. The plate was then gently shaken, covered, and incubated on a shaker at room temperature for 30 min. Absorbance was read at 440–460 nm using the ELISA microplate reader (Asys UVM 340, Wolf Laboratories Limited, York, UK). SOD activity Unit, defined as the quantity of enzyme needed to cause 50% dismutation of the superoxide radical and the SOD activity, was expressed as per gram tissue (Zakaria et al. 2015).

### Catalase

The liver tissue CAT activity was measured using the standard kit (Cayman Chemical, Ann Arbor, MI) according to the manufacturer’s protocol. Briefly, 20 μL of formaldehyde standard, CAT formaldehyde standard (positive control) at 0, 5, 15, 30, 45, 60, or 75 μM, or samples were added to the respective well of a 96-well plate followed by 30 μL methanol and 100 μL diluted assay buffer. To each of these wells, approximately 20 μL of diluted hydrogen peroxide were added to initiate the reaction. The plate was covered and incubated on a shaker at room temperature for 20 min. Approximately 30 μL diluted potassium hydroxide were added to each well, to stop the reaction, followed by 30 μL of CAT purpald (chromogen). The plate was incubated on a shaker at room temperature for 10 min. Finally, approximately 10 μL of CAT potassium periodate were added to each well and the plate was incubated on a shaker at room temperature for another 5 min. The absorbance was determined at 540 nm using the ELISA microplate reader (Asys UVM 340, Wolf Laboratories Limited, York, UK). Catalase activity was the quantity of enzyme that caused formation of 1.0 nmol formaldehyde per min at 25 °C and expressed as unit per gram tissue (Zakaria et al. 2015).

### Nitric oxide

Nitric oxide (NO) concentration was spectrophotometrically estimated in the liver homogenate using kits (eBioscience, San Diego, CA). The homogenate was incubated with absolute ethanol for 48 h to precipitate protein. Following centrifugation at 10,000 rpm for 5 min, the supernatants were incubated with 200 mL vanadium trichloride (8 mg/mL in 1 N HCl), and then Griess reagent was added. The mixture was then incubated at 37 °C for 30 min, cooled, and the absorbance was determined at 540 nm. The results were expressed as nmol/g of wet tissue.

### Interleukin-1ß, interleukin-6, and tumour necrotic factor

The concentrations of IL-1β, IL-6, and TNF-α in the supernatant from homogenized liver tissue were measured using ELISA kits (eBioscience, San Diego, CA) according to the manufacturer’s instructions (Liu et al. 2016; Zhang et al. 2017). Briefly, a capture antibody was added to each well of a 96-well plate and incubated overnight. Then, biotinylated antibody was added and incubated with supernatants or standard antigens. Finally, streptavidin was added to end the reaction, and the absorbance recorded at 450 nm. The results are expressed in picograms per milligram of protein (pg/mg) for cytokine concentrations.

## Histopathological analyses

Liver tissue sample from each rat was fixed in 10% formalin, embedded in paraffin, and then sectioned to 3–5 µm thick specimen. Each specimen was then stained with haematoxylin and eosin prior to the microscopic examination. The severity of liver tissue lesions was classified according to method described by Heikal et al. (2013) with slight modifications. Histopathological changes were scored as follows: (−) absent; (+) mild; (++) moderate, and (+++) severe.

## Phytochemicals analyses of MMCL

### UHPLC–ESI and GCMS analyses of MMCL

The UHPLC-ESI and GCMS analyses of MMCL were performed according to their procedure described elsewhere (Abdul Rahim et al. 2016). Briefly, the UHPLC-ESI analysis was performed using the Dionex 3000 UHPLC system (Thermo Fisher Scientific, Waltham, MA) whereas the chromatographic separation was performed using the BEH C18 UHPLC column (100 mm × 2.5 µm × 1.7 µm) maintained at 40 °C (Waters, Milford, MA). The flow-rate used was 0.3 mL/min and the injection volume was 10 µL of mobile phases comprising 0.1% formic acid in water (solution A) and 0.1% formic acid in acetonitrile (solution B). The gradient was initiated with 10% mobile phase B that reached 20% at 5 min. By 17 min, the gradient reached 60% mobile phase B before reaching the isocratic elution of 90% B after another 3 min. The gradient was held for 2 min for re-equilibration. The UHPLC system was coupled to a linear ion-trap-Orbitrap mass spectrometer Q Exactive from Thermo Fisher Scientific (Waltham, MA) supplied with an electrospray ionization (ESI) source. Instrument control and data acquisition were performed with Chameleon 6.8 software and Xcalibur 2.2 software (Thermo Fisher Scientific, Waltham, MA).

GC-MS analysis of MMCL was achieved using the Agilent 7890 A (Agilent Technologies, Santa Clara, CA) coupled with MSD quadrupole detector 5975 C (Agilent Technologies, Santa Clara, CA) (Abdul Rahim et al. 2016). The HP-5MS silica capillary column (30 m × 0.25 mm × 0.25 mm; Hewlett Packard) was used to separate the analytes in MMCL. An injection volume of 1 μL was employed (a split ratio of 1:10) with the carrier gas (helium gas; 99.999%) applied at a constant flow-rate of 1.0 mL/min. Total GC running time was 35.50 min. Mass spectra were taken at 70 eV at a scan interval of 0.5 s and fragments from 45 to 450 Da. The relative % of each component was calculated by comparing its average peak area to the total area. The software, Turbomass, used to handle mass spectra and chromatograms. Mass spectrum GC-MS interpretation was conducted using the database of National Institute Standard and Technology (NIST). The spectrum of the unknown components was compared with those of known components stored in the NIST library.

## Statistical analysis

All the data are presented as mean ± SEM. Statistical analysis was performed using GraphPad Prism version 5 (GraphPad Software, La Jolla, CA). Data obtained were analyzed using the one-way analysis of variance (ANOVA) and the differences between groups and the control group was determined using Dunnett’s *post hoc* test with *p* < 0.05 as the limit of significance.

## Results

### Hepatoprotective activity of MMCL

Table 2 shows the organ weight and body weight of rats with CCl_4_-induced liver damage pretreated with MMCL. The liver to body weight (LW/BW) was significantly (*p* < 0.05) higher in CCl_4_-treated and normal control group rats. Interestingly, pretreatment with MMCL significantly (*p* < 0.05) reduced LW/BW ratio in comparison with the CCl_4_-treated group in a dose-dependent manner.

**Table 2.** Effect of MEMC on body and liver weight in CCl_4_-treated rats.

Treatment	Dose (mg/kg)	Body Weight (BW) (g)	Liver Weight (LW) (g)	LW/BW (%)
Normal	–	204.1 ± 3.7	5. 6 ± 0.2	2.7 ± 0.1
10% DMSO + CCl_4_	–	208.9 ± 8.0	10.3 ± 0.3^a^	5.0 ± 0.2^a^
NAC + CCl_4_	50	190.2 ± 2.2	7.6 ± 0.4^ab^	4.0 ± 0.2^ab^
MEMC + CCl_4_	50	187.2 ± 3.1	7.9 ± 0.3^ab^	4.5 ± 0.2^a^
	250	205.8 ± 3.1	6.6 ± 1.0^ab^	3.2 ± 0.5^b^
	500	190.9 ± 3.3	6.6 ± 0.1^ab^	3.5 ± 0.1^b^

Values are expressed as means ± S.E.M. of six replicates.

a Significant different as compared with normal control, *p* < 0.05.

b Significant different as compared with negative control (10% DMSO + CCl_4_), *p* < 0.05.

## Blood liver enzymes

Table 3 shows the concentrations of plasma liver enzymes in rats with CCl_4_-induced liver damage pretreated with MMCL. The negative group showed significantly (*p* < 0.05) higher plasma ALT, AST, and ALP concentrations than the normal group. The plasma ALT and AST, but not ALP concentrations were significant (*p* < 0.05) lower only in rats pretreatment with 500 mg/kg body weight MMCL. On the other hand, NAC significantly (*p* < 0.05) reduced all plasma enzyme levels.

**Table 3.** Blood level of liver enzymes in hepatotoxic rats pretreated with MMCL.

Treatment	Dose (mg/kg)	ALT (U/L)	AST (U/L)	ALP (U/L)
Normal	–	15.8 ± 3.5	95.1 ± 7.3	102.7 ± 5.5
10% DMSO + CCl_4_	–	429.1 ± 81.5^a^	513.8 ± 12.0^a^	562.3 ± 36.8^a^
NAC + CCl_4_	50	191.7 ± 72.7^bc^	147.9 ± 27.2^bc^	216.7 ± 25.0^bc^
	50	634.1 ± 24.1^cd^	990.6 ± 39.7^cd^	566.7 ± 19.4^c^
MMCL + CCl_4_	250	333.0 ± 15.3^c^	723.1 ± 28.9^cd^	498.7 ± 68.4^c^
	500	168.7 ± 12.0^b^^c^	438.1 ± 16.3^bc^	454.7 ± 21.9^bc^

Values are expressed as means ± S.E.M. of six replicates.

a Significantly different when compared with the normal group following treatment with vehicle, *p* < 0.05.

b Significantly different when compared with negative control, *p* < 0.05.

c Significantly higher when compared with the normal group following treatment with test solutions, *p* < 0.05.

d Significantly higher when compared with negative control, *p* < 0.05.

ALT: alanine amino transaminase; AST: aspartate amino transaminase; ALP: alkaline phosphatase.

## Superoxide dismutase and catalase

Table 4 shows the SOD and CAT activities in liver tissue of rats with CCl_4_-induced liver damage pretreated with MMCL. From the results, the hepatotoxic group (negative) showed significantly (*p* < 0.05) lower SOD and CAT activities that the normal group. NAC and MMCL, at various doses, significantly (*p* < 0.05) reversed the effect of CCl_4_ on liver’s antioxidant enzymes by increasing the activities towards normal value.

**Table 4.** Effect of MMCL on antioxidant enzymes activity in liver tissue of CCl_4_-treated rats.

Treatment	Dose (mg/kg)	SOD (U/g tissue)	CAT (U/g tissue)
Normal	–	9.4 ± 0.8	122.3 ± 0.7
10% DMSO + CCl_4_		3.4 ± 0.2^a^	92.1 ± 1.0^a^
NAC + CCl_4_	50	8.7 ± 1.7^b^	121.7 ± 1.5^b^
MMCL + CCl_4_	50	6.1 ± 1.3^b^	116.9 ± 0.7^b^
	250	6.0 ± 0.4^b^	115.4 ± 2.0^b^
	500	5.5 ± 0.8^b^	114.4 ± 1.8^b^

Values are expressed as means ± S.E.M. of six replicates.

a Significant different as compared with normal control, *p* < 0.05.

b Significant different as compared with negative control (10% DMSO + CCl_4_), *p* < 0.05.

SOD: superoxide dismutase; CAT: catalase.

## Nitric oxide, tumour necrosis factor alpha, interleukin-1β, and interleukin-6

Table 5 shows the NO, TNF-α, IL-1β, and IL-6 concentrations in liver tissue of rats with CCl_4_-induced liver damage pretreated with MMCL. The NO concentration in the homogenates of CCl_4_-induced liver damage was significantly (*p* < 0.05) lower than in the normal liver. Interestingly, pretreatment with 250 and 500 mg/kg body weight MMCL significantly (*p* < 0.05) reversed the effect of CCl_4_ on NO level, causing the NO concentration to return to normal.

**Table 5.** Effect of MMCL on pro-inflammatory cytokines level in liver tissue of CCl_4_-treated rats.

Treatment	Dose (mg/kg)	NO (nmol/g tissue)	TNF-α (pg/mg protein)	IL-1β (pg/mg protein)	IL-6 (pg/mg protein)
Normal	–	18. 6 ± 2.6	29.7 ± 2.4	184.7 ± 11.8	21.5 ± 3.1
10% DMSO + CCl_4_	–	62.7 ± 11.8^a^	87.8 ± 3.7^a^	1474.4 ± 21.3^a^	136.7 ± 17.3^a^
MMCL + CCl_4_	50	53.5 ± 4.7	63.7 ± 4.1^b^	1167.1 ± 20.3^b^	98.2 ± 12.6^b^
	250	33.4 ± 5.1^b^	41.6 ± 2.0^b^	839.6 ± 14.9^b^	50.3 ± 8.7^b^
	500	24.1 ± 3.9^b^	32.7 ± 2.8^b^	618.3 ± 42.4^b^	30.8 ± 6.1^b^

Values are expressed as means ± S.E.M. of six replicates.

a Significant different as compared with normal control, *p* < 0.05.

b Significant different as compared with negative control (10% DMSO + CCl_4_), *p* < 0.05.

NO: nitric oxide; TNF-α: tumour necrosis factor alpha; IL-1β: interleukin 1β; IL-6: interleukin 6.

The hepatotoxic effect of CCl_4_ has led to significant (*p* < 0.05) higher TNF-α, IL-1β, and IL-6 concentrations in liver tissues of treated than normal. However, pretreatment with 250 and 500 mg/kg body weight MMCL caused significantly (*p* < 0.05) lower concentrations of these inflammatory in these liver tissues than those not treated.

## Histopathology

**Figure 1.**
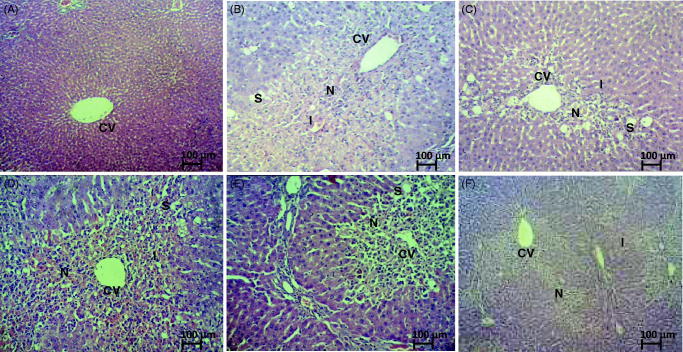
Microscopic analysis of liver section of normal and CCl_4_-induced hepatotoxic rats following pretreatment with 10% DMSO, NAC or MMCL. (A) Liver section of normal control animal exhibits normal hepatic cells each with well-defined cytoplasm, prominent nucleus and nucleolus and well brought central vein; (B) liver section of CCl_4_-induced hepatotoxic rats pretreated with 10% DMSO shows total loss of hepatic architecture with massive centrilobular hepatic necrosis, massive fatty changes vacuolization (steatosis), Kupffer cell hyperplasia and apoptosis; (C) liver section of CCl_4_-induced hepatotoxic rats pretreated with 50 mg/kg body weight of NAC demonstrates mild centrilobular hepatic necrosis, mark steatosis and mild haemorrhage; (D) liver section of CCl_4_-induced hepatotoxic rats pretreated with 50 mg/kg MMCL exhibits total loss of hepatic architecture with massive centrilobular hepatic necrosis, massive steatosis, kupffer cell hyperplasia, massive inflammation, massive haemorrhage and apoptosis; (E) liver section of CCl_4_-induced hepatotoxic rats pretreated with 250 mg/kg MMCL exhibits mark centrilobular hepatic necrosis, mild steatosis, marked inflammation, and mild haemorrhage; and (F) liver section of CCl_4_-induced hepatotoxic rats pretreated with 500 mg/kg MMCL shows normal liver architecture with mild centrilobular hepatic necrosis, mild inflammation (100× magnification). CV: central vein; N: necrosis; I: inflammation; S: steatosis.

The ability of MMCL to reverse the hepatotoxic effect of CCl_4_ was shown by the histopathological changes in the liver (Figure 1). Liver tissues of non-treated CCl_4_-induced hepatotoxic rats showed massive necrosis, marked inflammation, and mild steatosis (Figure 1(B)). Pretreatment with 50 mg/kg body weight of NAC prevented the development of these severe lesions, although the liver still showed centrilobular necrosis, steatosis, and haemorrhage (Figure 1(C)). The hepatoprotective effect of MMCL was only evident in rats treated with the highest dose of 500 mg/kg body weight used in the study. These rats had almost normal liver architecture, except for the mild centrilobular necrosis and inflammation (Figure 1(F) and Table 6).

**Table 6.** Histopathological scoring of the tissue of CCl_4_-induced hepatic injury rats after pretreatment with MMCL.

Treatment	Dose (mg/kg)	Steatosis	Necrosis	Inflammation	Haemorrhage
Normal	−	−	−	−	−
10% DMSO + CCl_4_		++	+++	+++	+
NAC + CCl_4_	50	+	+	+	+
	50	++	+++	+++	+++
MMCL + CCl_4_	250	+	++	++	++
	500	−	−	+	−

Histopathological score: (−) absent; (+) mild; (++) moderate, and (+++) severe.

## Phytochemicals analyses of MMCL

### UHPLC–ESI analysis of MMCL – identification of phenolic compounds in MMCL

**Figure 2.**
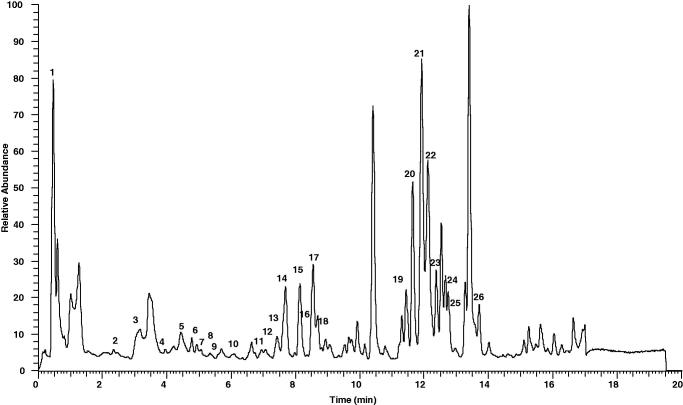
Total ion chromatography (TIC) of MMCL obtained using the UHPLC instrument in negative ion mode.

The UHPLC-ESI analysis showed that MMCL contains 26 phenolic compounds (Figure 2 and Table 7).

**Table 7.** Phenolic compounds identified in MMCL by UHPLC-MS.

Peak no.	*R*_T_ (min)	[M-H]-	Error (ppm)	Formula	Identification
1.	0.46	169.01392	5.090	C_7_H_5_O_5_	Gallic acid
2.	2.21	163.03985	5.394	C_9_H_7_O_3_	Protocatechuic acid
3.	3.16	193.05023	3.599	C_10_H_9_O_4_	Ferulic acid
4.	3.95	477.06860	4.681	C_21_H_17_O_13_	Quercetin-3-*O*-glucuronide
5.	4.58	599.10559	4.079	C_28_H_23_O_15_	Quercitrin-2″-O-gallate
6.	4.93	939.11462	5.125	C_41_H_31_O_26_	Pentagalloyl-hexoside II
7.	5.08	447.09439	4.926	C_21_H_19_O_11_	Kaempferol-3-*O*-galactoside
8.	5.24	317.03108	5.950	C_21_H_20_O_12_	Myricetin
9.	5.39	463.08957	5.392	C_15_H_9_O_8_	Quercetin-3-*O*-galactoside
10.	6.14	193.05037	4.324	C_10_H_9_O_4_	Isoferulic acid
11.	6.96	583.11093	4.576	C_28_H_23_O_14_	Afzelin-O-gallate
12.	7.37	301.03571	4.753	C_15_H_9_O_7_	Quercetin
13.	7.44	603.07947	4.209	C_30_H_19_O_14_	Quercetin dimer
14.	7.69	255.06647	5.037	C_15_H_11_O_4_	Pinocembrin (isomer 1)
15.	8.12	593.13110	3.596	C_30_H_25_O_13_	Kaempferol-3-*O*-glucoside
16.	8.14	315.05182	6.002	C_16_H_11_O_7_	Rhamnetin
17.	8.57	271.06100	3.321	C_15_H_12_O_5_	Pinobaksin
18.	8.94	285.04059	4.299	C_15_H_9_O_6_	Kaempferol
19.	11.45	255.06644	4.919	C_15_H_11_O_4_	Pinocembrin (isomer 2)
20.	11.64	253.05086	5.235	C_15_H_9_O_4_	Chyrsin
21.	11.92	299.05606	3.496	C_16_H_11_O_6_	Kaempferide I
22.	12.12	269.04529	3.123	C_15_H_9_O_5_	Genistein
23.	12.39	299.05649	4.934	C_16_H_11_O_6_	Kaempferide II
24.	12.75	313.07236	5.415	C_17_H_13_O_6_	Ermanin I
25.	12.78	313.07214	4.713	C_17_H_13_O_6_	Ermanin II
26.	13.71	269.08181	3.771	C_16_H_13_O_4_	Pinostrobin

### GC-MS analysis of MMCL – identification of volatile compounds in MMCL

**Figure 3.**
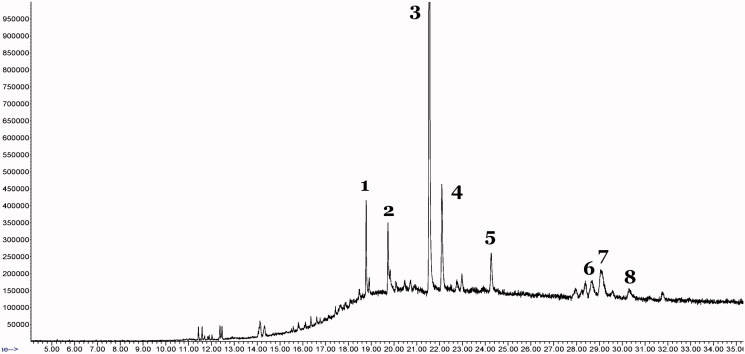
GCMS profile of MMCL revealed the presence of several peaks. Eight of these peaks belong to volatile compounds namely (**1**) methanone, 6-phenanthridinylphenyl-, oxime, (*E*)- (retention time (*R*_T_) = 18.78 min), (**2**) erucylamide/13-docosenamide, (*Z*) (*R*_T_ = 19.74 min), (**3**) 1,1′:3′,1″-terphenyl-4′,6′-dimethoxy-2,2″,3,3″,5,5″,6,6″-octamethyl- (*R*_T_ = 21.55 min), (**4**) cholest-4-en-3-one, 2-hydroxy-, (2α)- (*R*_T_ = 22.10 min), (**5**) α-tocopherol (*R*_T_ = 24.26 min), (**6**) 2-(acetoxymethyl)-3-(methoxycarbonyl) biphenylene (*R*_T_ = 28.64 min), (**7**) 6-methyl-2-phenylindole (*R*_T_ = 29.11 min), and (**8**) 5-methyl-2-phenylindolizine (*R*_T_ = 30.30 min).

The GC-MS profile of MMCL is shown in Figure 3. Several peaks were detected with, at least eight identified to be (**1**) methanone, 6-phenanthridinylphenyl-, oxime, (E)- (retention time (*R*_T_) = 18.78 min), (**2**) erucylamide/13-docosenamide, (Z) (*R*_T_ = 19.74 min), (**3**) 1,1′:3′,1″-terphenyl-4′,6′-dimethoxy-2,2″,3,3″,5,5″,6,6″-octamethyl- (*R*_T_ = 21.55 min), (**4**) cholest-4-en-3-one, 2-hydroxy-, (2α)- (*R*_T_ = 22.10 min), (**5**) α-tocopherol (*R*_T_ = 24.26 min), (**6**) 2-(acetoxymethyl)-3-(methoxycarbonyl) biphenylene (*R*_T_ = 28.64 min), (**7**) 6-methyl-2-phenylindole (*R*_T_ = 29.11 min), and (**8**) 5-methyl-2-phenylindolizine (*R*_T_ = 30.30 min) (Table 8).

**Table 8.** GCMS analysis of MMCL.

No. of peak	Retention time (min)	Name of the compound
1	18.783	Methanone, 6-phenanthridinylphenyl-, oxime, (*E*)
2	19.738	13-Docosenamide, (*Z*)
3	21.552	1,1′:3′,1″-Terphenyl, 4′, 6′-dimethoxy-2,2″,3,3″,5,5″,6,6″-octamethyl-
4	22.100	Cholest-4-en-3-one, 2- hydroxy-, (2.alpha.)-
5	24.264	Alpha-tocopherol
6	28.637	2-(Acetoxymethyl)-3-(methoxycarbonyl)biphenylene
7	29.110	6 Methyl-2 phenylindole
8	30.298	5-Methyl-2-phenylindolizine

## Discussion

The liver is primarily involved in detoxification and excretion of circulating compounds, which include toxic chemicals, as a mechanism in the preservation of health. Toxic chemicals are metabolized by the liver with subsequent release of liver tissue-damaging reactive oxygen species (ROS) or reactive nitrogen species (RNS) resulting in the leakage of liver enzymes into circulation. CCl_4_, classified as one of the volatile organic compounds (VOCs), is a lipophilic chemical and is highly toxic to the liver. Orally administered CCl_4_ is extensively absorbed in the gastrointestinal tract and the systemic absorption occurs very rapidly (Kim et al. 1990a). With regard to its mechanisms of toxicity, CCl_4_ is metabolized in the endoplasmic reticulum via CYP2E1 to form highly reactive metabolites, the trichloromethyl and trichloromethyl peroxy free radicals. These free radicals, in the presence of oxygen, bind covalently to cellular macromolecules particularly the polyunsaturated fatty acids of the membrane phospholipids, triggering lipid peroxidation and disrupting mitochondrial, endoplasmic reticulum, and plasma membranes. The reaction results in loss of membrane integrity, glucose-6-phosphatase activation, cellular calcium sequestration and homeostasis, reduction of protein synthesis, and leakage of microsomal enzymes, which contribute to cell damage. Other by-products of lipid peroxidation, including reactive aldehydes, also cause hepatotoxicity (Weber et al. 2003). In an attempt to assess the health risk inflicted by ingestion of CCl_4_-contaminated water, the previous toxicity studies use different types of digestible oils, as vehicle or diluent, to dissolve the volatile, lipophilic CCl_4_ prior to its oral administration to animals due to its poor solubility in water (Kim et al. 1990b). Once thought to be inert, vegetable oils in particular have been shown to produce physiological effects as well as to alter the absorption, target organ dose, and toxicity of CCl_4_. According to a study by Kim et al. (1990a), CCl_4_ given orally in four different ways, namely (i) in corn oil; (ii) as an aqueous emulsion; (iii) as the pure, undiluted chemical; or (iv) in saturated water, exerts different pharmacokinetics potential. The blood CCl_4_ concentration (*C*_max_) of equivalent level reached its peak between 3.5 and 6 min post-dosing when ingested as an aqueous emulsion or in water suggesting that the chemical is very rapidly and extensively absorbed from the gastrointestinal tract, and handled similarly by the body. These peak levels were higher than those in the undiluted CCl_4_ group and significantly higher than those in the corn oil group suggesting that corn oil markedly delayed the absorption of CCl_4_ from the gastrointestinal tract. Moreover, the higher bioavailability of CCl_4_ in aqueous emulsion and in water indicated by the highest *C*_max_ with the shortest *T*_max_ correlates well with the degree of hepatotoxicity. Based on these observations, it was suggested that the use of aqueous vegetable oil emulsion is more suitable in acute hepatotoxicity studies of CCl_4_ or other VOCs since the emulsion did not significantly alter the toxicity of CCl_4_ in comparison to the undiluted CCl_4_ or CCl_4_ ingested in water. In addition, it is often essential to utilize the aqueous emulsion preparations to give toxicologically relevant doses in an aqueous dosage form, owing to the limited solubility of CCl_4_ in water. Further studies carried out by Kim et al. (1990b) revealed the ability of dosing vehicles to extensively influence the acute hepatotoxic effect of CCl_4_ in rats. Using CCl_4_ in corn oil, in water, as an aqueous emulsion or in pure, undiluted form, the serum sorbitol dehydrogenase (SDH), glutamic-pyruvic transaminase (GPT), and ornithine carbamyl transferase (OCT) activities of the hepatotoxic liver were found to be elevated with the same time course, peaking at 48 h and diminishing considerably by 72 h. However, the serum enzyme activities in group that received CCl_4_ in corn oil were the lowest in comparison with the other groups suggesting a less hepatotoxic effect resulting from a delayed/prolonged systemic absorption and bioavailability of CCl_4_ caused by corn oil. The delayed/prolonged effects of corn oil on absorption and bioavailability of CCl_4_ are attributed to the oil ability to reduce intragastric motility and delay gastric emptying by acting as a reservoir in the gut to retard the systemic absorption of lipophilic chemicals conceivably delaying uptake of CCl_4_. In the present study, CCl_4_ was dissolved in 50% olive oil in water and was found to successfully induced hepatotoxicity when given orally to rats, thus, supported the previous reports that the emulsion-based vehicle did not significantly alter the pharmacokinetics or hepatotoxicity of CCl_4_ (Kim et al. 1990a, 1990b).

It is also worth-mentioning that the ability of a single dose of CCl_4_ in 50% olive oil used in the present study to cause an abrupt increase in liver size, LW/BW ratio and increase in serum liver enzymes is in accordance with a previous report made by Uemitsu and Nakayoshi (1984) and Kim et al. (1990b). These observations could be attributed to earlier finding that CCl_4_ in aqueous emulsion was absorbed rapidly and extensively from the gastrointestinal tract (Kim et al., 1990a) and reached its peak serum concentration (*C*_max_) between 3.5 and 6 min (shortest *T*_max_) after dosing in comparison to CCl_4_ dissolved in undiluted corn oil. Kim et al. (1990a) also cited earlier findings that absorption of radiolabeled CCl_4_ (^14^CCl_4_) into liver lipids and proteins, and hepatic lipoperoxidation reached its maximum within 5–90 min of intragastric administration of CCl_4_ to rats. Moreover, Kim et al. (1990b) also reported that the serum enzyme activities increase significantly 6 h after CCl_4_ dosing, reached peak concentration at 48 h and diminished considerably by 72 h. Based on this observations, it is plausible to suggest that during the first 6 h after CCl_4_ administration, the liver toxicity started to progress with maximum damage to the liver architecture at 48 h, which was indicated by an increase in liver weight. This was the reason why in the study design the animals were sacrificed 48 h after the administration of CCl_4_. Furthermore, the ability of CCl_4_ in aqueous emulsion to successfully triggered liver damage when administered as a single dose treatment could also be attributed to the use of animal model that was particularly sensitive to CCl_4_ hepatotoxicity. As cited by Kim et al. (1990b), the toxicity of CCl_4_ can be influenced by the age/body weight of rats whereby rats with lower body weight (200–250 g) were more susceptible to acute CCl_4_ hepatotoxicity than those weighing 300–350 g.

The liver is one of the organs most prone to damage caused by ROS and lipid peroxidation. Organisms have developed a sophisticated antioxidant system, primarily SOD and CAT, to protect the liver and maintain the redox homeostasis. However, excessive production of ROS can disrupt homeostasis and cause damage to the liver. MMCL was shown to possess remarkable antioxidant activities, primarily due its high content of phenolic compounds (Zakaria et al. 2014). In the current study, pretreatment of MMCL appeared to protect the liver from developing CCl_4_-induced liver damage. This hepatoprotective property of MMCL is partly thorough stimulation of liver tissue SOD and CAT activities and subsequently clearance of the liver tissue-damaging ROS.

Among effects of CCl_4_ on the liver is the initiation of inflammation in liver tissues. Like most tissues, liver inflammation manifest as release of inflammatory markers including NO, TNF-α, IL-1β, and IL-6. The markers are pro-inflammatory and mediate acute phase protein synthesis, lipid metabolism, cholestasis, and fibrosis (Sharma et al. 2007; Niederreiter and Tilg, 2018). Although still controversial, the role of NO in liver has been associated with both beneficial and detrimental consequences. For example, NO exerts a protective role in mild oxidative hepatotoxicity models. Moreover, previous study has also suggested that the hepatoprotective effects of NO seen in the CCl_4_-induced model might be partly due to the inhibition of TNF-α (Morio et al. 2001), which together with IL-1β and IL-6 constitute part of pro-inflammatory cytokines (Kandil et al. 2017). Various cytokine receptors, including those for TNF-α, IL-1β, and IL-6, can be found in normal hepatocytes and, upon chemical-induced injury to Kupffer cells, hepatic stellates, and sinusoid endothelial cells these pro-inflammatory cytokines are produce/release resulting in infiltration of neutrophils and monocytes into the damaged liver (Zhang et al. 2004). In the present study, CCl_4_ administration caused significant lower of liver NO concentration, but significantly increased the TNF-α, IL-1β, and IL-6 concentrations compared to normal. In rat with CCl_4_-induced hepatotoxicity, with MMCL pretreatment, the liver NO concentration increased and the TNF-α, IL-1β, and IL-6 concentrations decreased, suggesting that the extract have protective effects on liver from inflammation (Zakaria et al. 2007). The hepatoprotective effect of MMCL, particular at high treatment concentration, was also evident in the liver histopathological results of the study. At 500 mg/kg body weight MMCL, the treated rats did not develop the severe liver lesions seen in non-treated rats. These findings further supported previous claims that MMCL possesses anti-inflammatory activity (Zakaria et al. 2007; Balan et al. 2015).

In this study, we showed that the effect of MMCL in the amelioration of CCl_4_-induced hepatotoxicity is also reflected by the change in plasma liver enzymes. The plasma ALT, AST, and ALP concentrations increase following the CCl_4_ administration, indicating hepatocellular damage and disruption of structural integrity of the liver. These observations could be directly attributed to the ability of CCl_4_ to induce lipid peroxidation, which, in turn, caused hepatocytes membrane injury with subsequent necrosis as demonstrated in the histopathological analysis. Disruption of hepatocytes membrane integrity resulted in the dissolution of the cell contents, including the said enzymes, into blood circulation (Tolman and Dalpiaz 2013). Pretreatment with MMCL or NAC were able to reduce the ALT and AST, but not ALP levels supported the above connotation on the potential of MMCL to exert hepatoprotective effect. In the liver, ALT is localized solely in the cellular cytoplasm, whereas AST is found both in the cytosol and mitochondrial. Therefore, an increase in ALT is a more specific indicator for liver damage (Giannini et al. 2005). Although ALP is ubiquitous, found in various organs and cells of the body, including the liver, the source of plasma ALP, in this case, is presumably the liver. Moreover, the high level of ALP could also be attributed to its long half-life in the circulation (7 d) in comparison with the transaminases [ALT (47 h) and AST (17 h)] leading to its slow decrease after resolution (Giannini et al. 2005).

UHPLC-ESI analysis showed that MMCL contained at least 26 flavonoid-based bioactive compounds. Of these, 22 bioactive compounds were detected in the ethyl acetate partition of MMCL (Zakaria et al. 2016). Some of these compounds in MMCL, such as gallic acid, ferulic acid, quercetin, and genistein have been reported to protect the liver from CCl_4_-induced toxicity (Pavanato et al. 2003; Kuzu et al. 2007; Tung et al. 2009; Kim et al. 2011). Gallic acid, for example, downregulated CYP_2_E_1_ expression in liver tissues while increasing SOD activities. These effects serve to modulate the antioxidant enzymes activities and inhibit lipid peroxidation. Ferulic acid, on the other hand, attenuates the serum of TNF-α, iNOS, and COX-2 mRNA expressions. Ferulic acid also attenuated cytosolic NF-κβ, phosphorylated JNK, p38 mitogen-activated protein (MAP) kinase, and nuclear translocation of activated c-Jun, while decreasing the cytosolic concentrations of NF-κβ inhibitors and TLR_4_. Thus, in CCl_4_-induced liver damage, the ferulic acid in MMCL reduces the adverse effects caused by oxidative radicals and inflammatory response. The hepatoprotective activity of quercetin is through the reduction of liver collagen content, iNOS expression, and lipid peroxidation, which are associated with an improved total peroxyl radical-trapping antioxidant capacity. Genistein can attenuate liver toxicity by inhibiting lipid peroxidation and stimulating antioxidation. In addition, genistein also exerts anti-inflammatory and antinecrotic effects, which further contributes to the attenuation of liver toxicity. It is, therefore, reasonable to assume that the hepatoprotective activities of MMCL is attributed to its gallic acid, ferulic acid, quercetin, and genistein components that most possibly act synergistically (Morio et al. 2001).

## Conclusions

MMCL effectively prevent CCl_4_-induced liver toxicity in rats by attenuating lipid peroxidation, stimulating antioxidation through SOD and CAT, anti-inflammation through upregulation of NO and downregulation of TNF-α, IL-1β, and IL-6 activities. The hepatoprotective activities of MMCL are proposed to occur through the synergic effects of its chemical component, namely, gallic acid, ferulic acid, quercetin, and genistein.
